# Clinical Significance and Expression Pattern of RIP5 and VGLL4 in Clear Cell Renal Cell Carcinoma Patients Treated with Sunitinib

**DOI:** 10.3390/biomedicines12010149

**Published:** 2024-01-10

**Authors:** Tanja Tomić, Davor Tomić, Martina Vukoja, Marija Kraljević, Ivona Ljevak, Una Glamočlija, Vajdana Tomić, Katarina Vukojević, Renata Beljan Perak, Violeta Šoljić

**Affiliations:** 1 Faculty of Health Studies, University of Mostar, Bijeli Brijeg bb, 88000 Mostar, Bosnia and Herzegovina; tanja.tomic@fzs.sum.ba (T.T.); ivona.ljevak@fzs.sum.ba (I.L.); vajdana.tomic@fzs.sum.ba (V.T.); violeta.soljic@fzs.sum.ba (V.Š.); 2 Department of Urology, University Hospital Center Mostar, Bijeli Brijeg bb, 88000 Mostar, Bosnia and Herzegovina; sven.tomic@gmail.com; 3 Laboratory of Morphology, Department of Histology and Embryology, School of Medicine, University of Mostar, Bijeli Brijeg bb, 88000 Mostar, Bosnia and Herzegovina; martina.vukoja@mef.sum.ba; 4 Department of Oncology, University Hospital Center Mostar, Bijeli Brijeg bb, 88000 Mostar, Bosnia and Herzegovina; marija.kraljevicc91@gmail.com; 5 Faculty of Pharmacy, University of Sarajevo, Zmaja od Bosne 8, 71000 Sarajevo, Bosnia and Herzegovina; una.glamoclija@ffsa.unsa.ba; 6 Department of Gynecology, University Hospital Center Mostar, Bijeli Brijeg bb, 88000 Mostar, Bosnia and Herzegovina; 7 Department of Anatomy, Histology and Embryology, University of Split School of Medicine, Šoltanska 2, 21000 Split, Croatia; 8 Department of Pathology, Forensic Medicine and Cytology, University Hospital of Split, Spinčićeva 1, 21000 Split, Croatia; renatavonbe@gmail.com

**Keywords:** ccRCC, RIP 5, VGLL 4, sunitinib, progression-free survival, mRCC

## Abstract

While clear cell renal cell carcinoma (ccRCC) is curable, advanced metastatic (mRCC) remains a clinical challenge. We analyzed clinical, pathohistological, and molecular data (Receptor Interacting Protein 5—RIP5 and Vestigial Like Family Member 4—VGLL4 expression) of 55 mRCC patients treated with first-line treatment with sunitinib. The trend of linear increase in the protein expression of RIP5 was observed with the progression of tumor grade. Overall, 80% of RIP5-positive cells were in the control kidneys and high-grade mRCC. On the contrary, RIP5 displayed low expression in grade 2 mRCC (5.63%). The trend of linear decrease in the expression of VGLL4 was observed with the progression of tumor grade. The highest protein expression of VGLL4 was observed in grade 2 (87.82%) in comparison to grade 3 and 4 and control. High expression of RIP5 mRNA was associated with longer first-line overall survival and longer progression-free survival in mRCC. In addition, a high VGLL4 mRNA expression showed better overall survival in patients with ccRCC. In conclusion, high mRNA expression of RIP5 and VGLL4 are important markers of better survival rates in mRCC patients.

## 1. Introduction

Clear cell renal cell carcinoma (ccRCC) is the most common type of renal cell carcinoma, accounting for about 75 to 80% of renal cell carcinomas [1]. The remaining 20 to 25% are papillary renal cell carcinoma (pRCC), chromophobe RCC (cRCC), and others, while about 5% of renal tumors remain unclassified [2]. ccRCC is responsible for the death of 180,000 people a year worldwide [3]. ccRCC arises from the proximal epithelium of the renal tubules and is characterized by genetic diversity and chromosomal complexity. Loss of heterozygosity of chromosome 3p, where the von Hippel-Lindau (VHL) gene is located, is found in more than 90% of cases of ccRCC and is considered a critical genetic event [4,5,6]. Loss-of-function mutation in VHL gene induces abnormal regulation of a number of VHL-mediated targets, pathways, and processes, which is a significant step in the onset and development of ccRCC [7,8].

Prognostic factors are pathological stage, tumor grade, presence of tumor necrosis, and rhabdoid or sarcomatous differentiation. Today, a four-stage classification according to WHO/ISUP is used based on nucleolar prominence, nuclear pleomorphism, and/or rhabdoid, and/or sarcomatoid differentiation [9]. Surgical treatment is effective in localized disease in the early stage with partial or radical nephrectomy, and in advanced cancer or metastatic cancer treatment still represents a clinical challenge [10,11,12]. Namely, ccRCC is not sensitive to chemo and radiotherapy, and 45% of diagnosed cancers are metastatic, while 30% of operated patients develop disease relapse, including a stratified group of low-risk progressions [13,14,15,16,17]. Treatment with tyrosine kinase inhibitors, immunotherapy, inhibitors of the targeted pathway of immune checkpoints, provided patients with a significant extension of overall survival, and the absence of disease progression [18,19]. Namely, receptor tyrosine kinase inhibitor (sunitinib) is one of the most common treatment options. Sunitinib inhibits different targets such as the platelet-derived growth factor receptors and the vascular endothelial growth factor. In this capacity, sunitinib is used in the treatment of renal cell carcinoma and gastrointestinal stromal tumors [20]. However, the effectiveness of sunitinib is limited to 30% of patients who have a successful response to therapy in contrast to those who have a weak response with the development of long-term relapses [21,22,23,24]. Therefore, it is necessary to find novel predictive markers that will provide adequate response to given treatment.

Receptor Interacting Protein (RIP) kinases represent a family of dual serine/threonine and tyrosine kinases that play a key signaling role in cell survival and death [25]. Each member of the RIP family contains a conserved RIP kinase domain and other domains that determine a specific function through protein-protein interactions. It was mentioned for the first time in 2004, as a protein associated with Dusty protein kinase, and was investigated in human congenital anomalies of the kidney and urinary tract (CAKUT), and involvement in the regulation of the Fibroblast Growth Factor (FGF) signaling pathway [25,26]. In the fetal kidney, RIP5 is co-expressed in the metanephric mesenchyme and ureteral bud, making it a critical regulator of human urinary tract development downstream of fibroblast growth factor signaling [27,28,29,30]. Changes at the genetic, epigenetic, and expression level have been recorded in cancer cells. RIP5 is studied in various types of cancer, including colorectal cancer, lung adenocarcinoma, and triple-negative breast cancer [31,32,33]. RIP5 in colorectal cancer promotes metastasis and chemoresistance via epithelial-mesenchymal transformation (EMT) [34,35,36]. Therefore, current findings suggest that RIP kinases represent attractive targets for the development of improved treatments for certain types of cancer [37].

Vestigial Like Family Member 4 (VGLL4) is a transcriptional cofactor of the VGLL family, which includes VGLL1-4. VGLL proteins are considered a new group of TEAD interacting partners, as an active participant in tumor genesis and metastasis [38,39,40,41]. VGLL4 has been described as a tumor suppressor in many types of cancer, for example in lung cancer [38], breast cancer [42], stomach cancer [43,44,45], colorectal cancer [46], bladder cancer [47,48], adenocarcinoma of the pancreas [49], and squamous carcinoma of the esophagus [50]. The tumor suppressor function of VGLL 4 has been demonstrated; however, the regulation of VGLL 4 remains elusive. Lower expression of VGLL 4 usually indicates poor survival. VGLL4 partially inhibits EMT through suppression of the vWnt/beta-catenin signaling pathway [44]. Therefore, the aim of this study is to investigate expression of RIP5 and VGLL 4 in metastatic ccRCC (mRCC) in the context of possible predictive markers for this deadly disease.

mRCC is a significant health concern, with a poor prognosis and a growing need for more effective treatment options. The current management of mRCC is rapidly evolving. Recent advances in targeted therapies and a better understanding of the disease’s biology have led to improvements in patient care and prognosis. Understanding the latest advances in diagnosis and management can assist oncologists and nephrologists in better diagnosing and treating mRCC patients.

## 2. Materials and Methods

### 2.1. Patient Data

The study was approved by the Institutional Review Board. Informed consent was obtained from all patients. A total of 55 mRCC patients had a radical nephrectomy between 2009 and 2019 at the University Hospital Mostar, Bosnia, and Herzegovina. The clinical characteristics were used from the patient records within hospital information system. Enrolled patients had histopathological diagnosis of ccRCC and were presented with metastatic disease initially or during follow-up. We excluded patients who received prior targeted therapies, patients with concurrent chronic renal disease, and incomplete clinical data. A total of 45 patients were enrolled. These patients received sunitinib as a first-line treatment. Patients received an oral dose of sunitinib 50 mg daily for 4 weeks. Patients were regularly re-evaluated, usually after 2–3 therapeutic cycles.

### 2.2. Tissue Procurement and Processing

Formalin-fixed paraffin-embedded (FFPE) samples from patients diagnosed with mRCC were recruited from the Department of Pathology, University Hospital Centre Mostar. A total cohort included 55 samples, including 45 samples from patients diagnosed with mRCC. Out of 55 samples, 34 samples underwent genetic expression analysis. Tissue samples from mRCC were fixed with 4% xylol, dehydrated in graded ethanol dilutions, embedded in paraffin blocks, serially cut (4 μm), and mounted on glass slides. 

### 2.3. Double Immunofluorescence

All tissue sections were deparaffinized in xylol, dehydrated in graded ethanol dilutions, and washed three times in desilted water. The sections were heated in citrate buffer for antigen retrieval for 15 min. After cooling, tissue sections were incubated with the combination of primary antibodies RIP5 (N-16) (diluted 1:50, Santa Cruz Biotechnology, Santa Cruz, CA, USA, sc 162109) and VGLL4 (diluted 1:50, Invitrogen, Waltham, MA, USA PA5-58467) overnight at +4 °C. Primary antibodies were washed in PBS three times and incubated with a combination of secondary antibodies: Goat Anti-Rabbit IgG H&L (Abcam, Cambridge, UK, Alexa Fluor^®^ 594) (ab150080) and Donkey Anti-Goat IgG H&L (Abcam, Cambridge, UK, Alexa Fluor^®^ 488) (ab150129) in Dako Antibody Diluent (Dako, Glostrup, Denmark, S0809) for 1 h at room temperature. After incubation with secondary antibodies, sections were washed in PBS three times, counterstained with DAPI, and coverslipped (Immuno-mount, Shandon Inc., Pittsburgh, PA, USA).

### 2.4. Sample Processing and RNA Isolation

RNA was isolated from 34 FFPE mRCC samples, and healthy border renal tissue from the same patient as a control. GenElute™ FFPE RNA Purification Kit (Sigma-Aldrich, Taufkirchen, Germany) was used for RNA isolation, as previously described [51]. Concentration of RNA was measured by Qubit™ 4 Fluorometer (Thermo Fisher Scientific Inc. Waltham, MA, USA).

### 2.5. qPCR

After RNA isolation, an iTaq™ Universal SYBR^®^ Green One-Step Kit (Bio-Rad, Hercules, CA, USA) designed for measurement of gene expression was used. The master mix containing iTaq™ Universal SYBR^®^ Green reaction mix, iScript reverse transcriptase, selected forward and reverse primers (Table 1), nuclease-free H_2_O, and RNA was mixed in a 96-well plate. All samples were prepared in duplicate, and RPL13a was used as the reference gen. The negative control contained a master mix without RNA. The plate was analyzed using the Applied Biosystems TM 7500 RT-PCR system (Thermo Fisher Scientific, Waltham, MA, USA). The 2^−∆∆^Ct method was used to calculate the relative fold gene expression of samples.

### 2.6. Transcriptomics

We extracted the cancer tissue ribonucleic (RNA)-sequencing data in terms of fragments per kilobase of transcript per million mapped reads upper quartile (FPKM-UQ) from public data resources—The University of California Santa Cruz cancer browser UCSC Xena (xenabrowser.net). Overall survival and RIP5 (DSTYK) and VGLL4 gene expression data from the Genomic Data Commons—The Cancer Genome Atlas Kidney Renal Clear Cell Carcinoma (GDC TCGA KIRC) studies were exported and edited in Microsoft^®^ Excel^®^ 2019 MSO version 2305 (Microsoft Corp., Redmond, WA, USA). After the double samples were excluded from data curation, 526 patients were included in the survival analysis for ccRCC.

### 2.7. Statistical Analysis

Overall survival (OS) was defined as the duration from the date of surgery to death or last follow-up. Disease-free survival (DFS) was defined as the duration from the date of surgery to date of distant metastasis. Progression-free survival (PFS) was defined from the start of sunitinib therapy to the date of the progression or last follow-up. In the statistical analysis, descriptive statistics was used. Kaplan–Meier survival curves were prepared with the Log-rank (Mantel–Cox) test, Breslow (Generalized Wilcoxon) test, and Tarone–Ware test for comparison between patients in two groups (<1 RIP5 fold gene expression and >1 RIP5 fold gene expression for data from our experiment and high and low expression of RIP5 and VGLL4 from GDC TCGA KIRC). The Mantel–Cox proportional hazard regression model was applied with age, RIP5 fold gene expression group, and TNM stage of disease. Accepted statistical significance was at the *p* < 0.05 level. Statistical analysis was performed using the SPSS (Statistical Package for Social Sciences) program version 23.0 and using R Statistical Software (Foundation for Statistical Computing, Vienna, Austria) version 4.3.2. packages “survival”, “survminer”, and “My.stepwise”.

## 3. Results

The demographic and clinical characteristics of patients are displayed in Table 2. There were 34 patients with median age of 61 years (interquartile range (IQR) 55–67). Median time from sunitinib prescription to treatment initiation was 2 months (IQR 0 3). Median time from mRCC diagnosis to sunitinib initiation was 3 months (IQR 2–4).

### 3.1. RIP5 and VGLL4 Protein Expression

**Figure 1:**
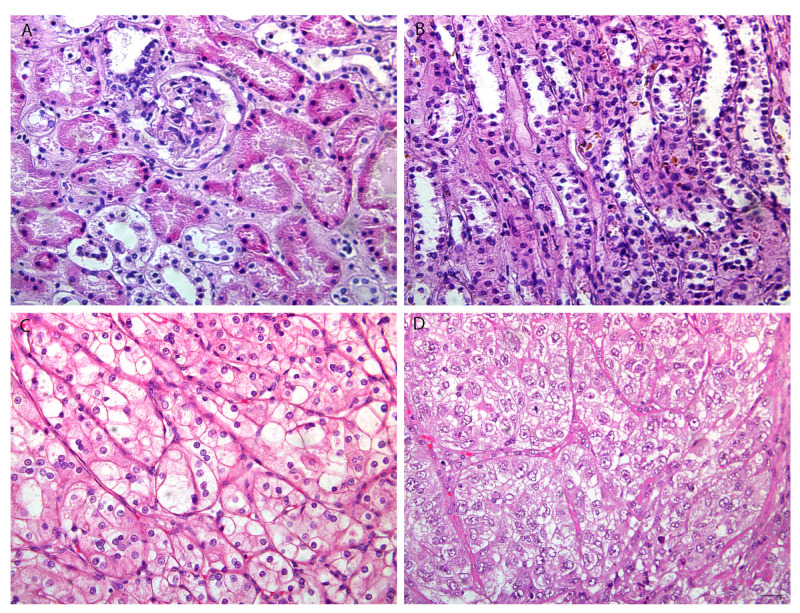
The basic morphology of control renal tissue and mRCC. Normal (CTRL) kidney cortex (**A**) and medulla (**B**) and metastatic renal cell carcinoma (mRCC) grade 2 (G2) (**C**), and grade 4 (G4) (**D**). Scale bar 25 µm. Hematoxylin and eosin staining.

In this study, we investigated mRCC in patients with different tumor grades. In order to show double immunofluorescence staining with RIP5 and VGLL4, we firstly wanted to present the basic morphology (Figure 1) of mRCC and control renal tissue. Epithelial cells of proximal tubules are the origin site of metastatic renal cell carcinoma (mRCC). Different grades of mRCC display a wide range of morphological changes, characterized from small nuclei with inconspicuous nucleoli in grade 2 to nuclear polymorphism and rhabdoid or sarcomatoid features in grade 4 (Figure 1).

**Figure 2:**
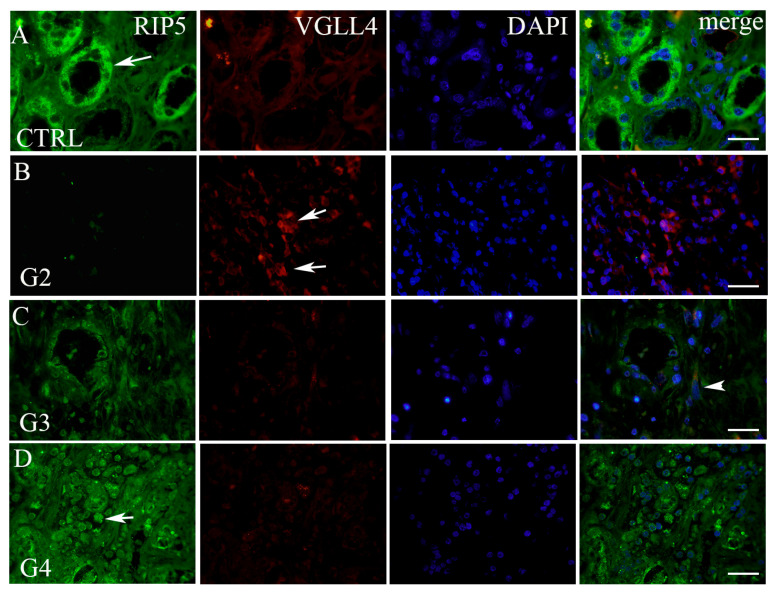
Double immunofluorescence with RIP5, VGLL4, and DAPI. RIP5 (green)- and VGLL4 (red)-positive cells (arrows—positive cells, regardless of staining intensity) in the normal kidney (CTRL) cortex (**A**) and different grades of mRCC: grade 2—G2 (**B**), grade 3—G3 (**C**), and grade 4—G4 (**D**). Co-localization of RIP5 and VGLL4 (arrowhead); merge (all panels in the row combined). Immunofluorescence staining with RIP5, VGLL4, and DAPI. Scale bar 25 µm.

By double immunofluorescence staining with RIP5 and VGLL4, we wanted to point out how many cells and which cells in mRCC and control kidney tissue express these two proteins. In the renal cortex and medulla of normal control kidneys, RIP5 was expressed in both glomeruli and tubules (Figure 2), with strong diffuse expression in epithelial cells (Table 3). Overall, 80% of RIP5-positive cells were in the control kidneys (Figure 2A). This was also the highest expression of RIP5 in comparison to different mRCC grades, especially grade 3 and 4 (*p* ˂ 0.0001 and *p* ˂ 0.01, respectively) (Figure 2A).

**Figure 3:**
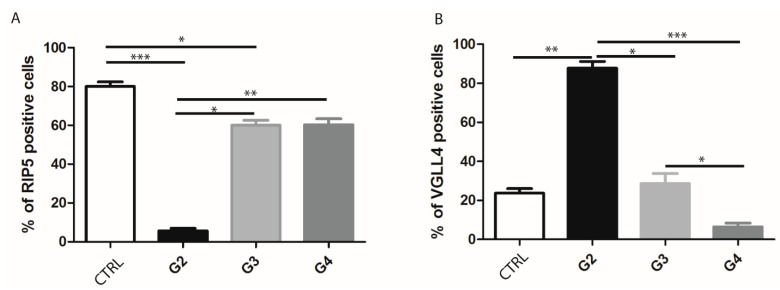
Ratio of RIP5- and VGLL4-positive cells in control kidney and mRCC. Graphic presentation of quantitative expression of RIP5 (**A**), and VGLL4 (**B**) in control (CTRL) and different grades of mRCC (G2–G4). Data are presented as mean ± SD. * *p* ˂ 0.01, ** *p* ˂ 0.001, *** *p* ˂ 0.0001, Kruskal–Wallis followed by Dunn’s multiple comparison test.

On the contrary, RIP5 displayed low expression in grade 2 mRCC (Figure 2, Table 3), with 5.63% of positive cells (Figure 3A). Grade 3 and 4 had similar number of RIP5-positive cells (60%) (Figure 3A) and strong expression in the epithelial cells (Table 3). The lowest expression of RIP5 was observed in grade 2, being lower than grades 3 and 4 (*p* ˂ 0.01 and *p* ˂ 0.001, respectively). The trend of linear increase in the expression of RIP5 was observed with the progression of tumor grade (*p* ˂ 0.001).

We performed a double immunofluorescence staining with VGLL4 to determine whether there is a co-expression with RIP5. RIP5 and VGLL4 double-positive cells were observed only in grade 3 tumors. In the renal cortex and medulla of normal control kidneys, VGLL4 is expressed in tubules (Figure 2) with strong focal expression in epithelial cells (Table 3). The percentage of VGLL4-positive cells in the control kidneys was 23.7% (Figure 3B), which was statistically different only in comparison to grade 2 (*p* < 0.001) (Figure 3B). Additionally, the highest expression of VGLL4 was observed in grade 2, with 87.82% of positive cells in comparison to grade 3 and 4 (*p* < 0.01 and *p* ˂ 0.0001, respectively) (Figure 3B). VGLL4 displayed low expression in grade 4 mRCC (Figure 2D, Table 3), with 6.45% of positive cells (Figure 3B). In grade 3 there was 28.64% of VGLL4-positive cells (Figure 3B), with moderate expression and focal staining in the epithelial cells (Table 3). In addition, grade 3 displayed statistically higher expression in comparison to grade 4 (*p* < 0.01). The trend of linear decrease in the expression of VGLL4 was observed with the progression of tumor grade (*p* < 0.0001).

### 3.2. Gene Expression Analysis

**Figure 4:**
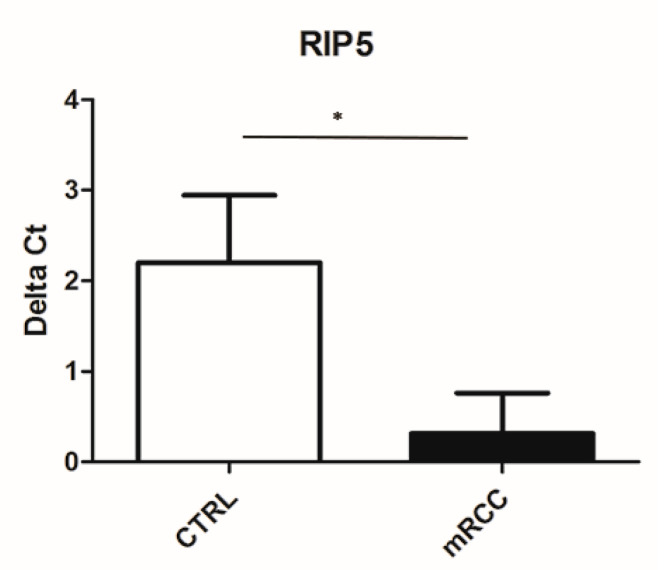
Ct value of RIP5 in control kidney and mRCC. qPCR was performed to determine the mRNA levels of RIP5 in metastatic renal cell carcinoma and control tissues. Higher Ct value indicated lower gene expression. * *p* ˂ 0.05.

In order to further investigate mRNA expression of RIP5 and VGLL4 in our specimens, we performed the qPCR method on our samples. We detected the mRNA of RIP5 in 34 mRCC cancer tissues and 34 control tissues by qPCR. The mRNA level of RIP5 were higher in mRCC cancer in comparison to the control (Figure 4).

**Figure 5:**
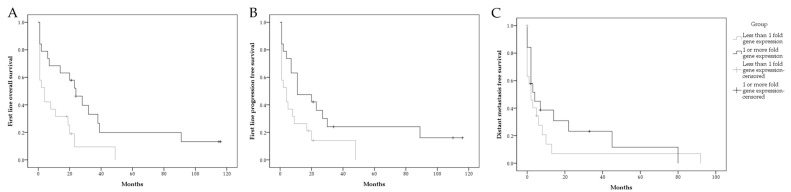
Graphical presentation of survival in mRCC patient. First-line overall survival stratified by *RIP5* fold gene expression (**A**); first-line progression-free survival stratified by *RIP5* fold gene expression (**B**); distant metastasis free survival stratified by *RIP5* fold gene expression (**C**).

RIP5 low expression (fold gene expression below one) was significantly associated with shorter first-line overall survival and progression-free survival in our cohort (Table 4, Figure 5A,B).

The distant metastasis-free survival between groups was not statistically significant (Table 4, Figure 5C).

**Figure 6:**
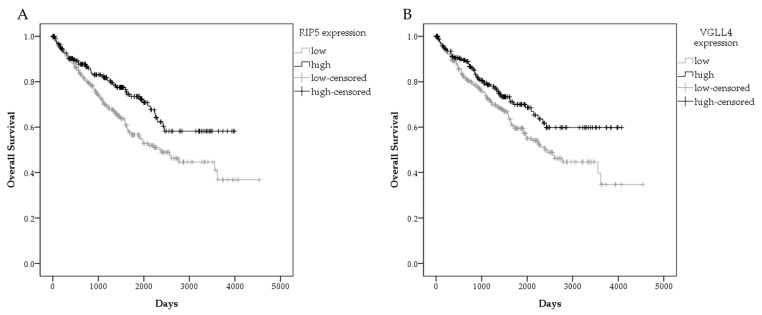
Graphical presentation of survival in ccRCC patient in GDC TCGA KIRC. Overall survival by gene expression RNAseq-HTSeq-FPKM-UQ for patients with high and low *RIP5* expression (**A**), and high and low *VGLL4* expression (**B**). Data used from the GDC TCGA KIRC.

In order to prove consistency of our results on mRNA expression in mRCC and our data of patient survival, we additionally performed analysis of patients survival in ccRCC patients in a GDC TCGA KIRC study. Similarly, low RIP5 gene expression was associated with significantly lower mean overall survival in 526 patients from GDC TCGA KIRC. The mean overall survival with 95% confidence interval (CI) in a group with low RIP5 expression was 2597 days (2341–2853), while in a group with high RIP5 expression, it was 2866 days (2617–3115) (Figure 6). All applied statistical tests indicated significant differences, with *p*-values 0.003, 0.011, and 0.005 for Log-rank (Mantel–Cox), Breslow (Generalized Wilcoxon), and Tarone–Ware tests, respectively.

In addition, low expression of VGLL4 was analyzed. Although the Breslow (Generalized Wilcoxon) test indicated no significant differences between VGLL4 expression and overall survival (*p* = 0.058), Log-rank (Mantel–Cox) and Tarone–Ware tests indicated there was a worse overall survival in patients with low VGLL4 expression (*p* = 0.018 and *p* = 0.037, respectively). Mean overall survival with 95% confidence interval (CI) in the group with low VGLL4 expression was 2609 days (2349–2869) compared to the group with high VGLL4 expression with 2905 days (2649–3161) (Figure 6B).

In a Mantel–Cox proportional hazard regression model, proportional hazards assumption was confirmed by evaluation of independence of Schoenfeld residuals and time (global *p* = 0.84, age *p* = 0.38, TNM *p* = 0.65, fold-gene expression group *p* = 0.71). Stepwise variable selection procedure resulted in the final model including the fold-gene expression group, and was significant with *p*-value 0.03. RIP5 gene expression higher than one-fold was found to be independently, significantly associated with lower hazard risk for death when evaluating overall survival (Table 5). In patients with RIP5 gene expression higher than one-fold, death risk was decreased by 58% compared to patients with RIP5 gene expression lower than one-fold (Table 5).

## 4. Discussion

ccRCC is curable in the early localized stage by partial or total surgical nephrectomy, while advanced mRCC remains a clinical challenge [12]. Additionally, due to the high level of tumor heterogeneity in mRCC, there is a need to find new treatment options for a precise personalized medicine approach to tackle this deadly disease. Integrated molecular analysis of mRCC identified several gene expression signatures associated with tumor progression and poor prognosis [42]. Alterations of RIP5 kinases at expression levels are frequently found in high number of cancers, and this might suggest that RIP5 kinases promote tumor progression and metastasis. However, there is a lack of literature that points to mechanistic involvement of RIP5 kinases in either a pro-tumor or anti-tumor manner, in regard to specific cancer. Additionally, contributions of RIP kinases as a therapeutical target have been shown useful in clinical trials in inflammatory diseases [37]. Therefore, RIP kinases expression in mRCC might represent an attractive therapeutic target.

In the renal cortex and medulla of normal control kidneys, RIP5 was expressed in both glomeruli and tubules, with strong diffuse expression in epithelial cells. There was 80% of RIP5-positive cells in the control kidneys. This was also the highest expression of RIP5 in comparison to different mRCC grades, especially grade 3 and 4. On the contrary RIP5 displayed low expression in grade 2 mRCC. Grade 3 and 4 had a similar number of RIP5-positive cells (60%), and strong expression in the epithelial cells. The lowest expression of RIP5 was observed in grade 2, being lower than grades 3 and 4. The trend of linear increase in the expression of RIP5 was observed with the progression of tumor grade. This finding is in accordance with Cohen et al. that showed how grade 3 and 4 generally have lower mean overall survival [52]. In our study in mRCC, high expression of RIP5 mRNA was associated with longer first-line overall survival and longer progression-free survival (the time from sunitinib initiation until disease progression or worsening). However, we need to take into account different results between mRNA and protein levels in our study because this might be attributed to low consistency between RIP5 antibody staining and RIP5 RNA expression data, as suggested in the Human Protein Atlas proteinatlas.org (accessed on 11 November 2023). Additionally, different results between mRNA and protein levels can be explained by the fact that sunitinib is a moderate inhibitor of dual serine/threonine and tyrosine kinases [53,54]. Accordingly, patients in our study that had higher expression of RIP5 and received sunitinib had longer progression-free survival. Similarly, when analyzing patients from GDC TCGA KIRC, high RIP5 gene expression was associated with significantly longer mean overall survival. Zhong et al. also showed that lower RIP5 expression accelerates lung cancer cell growth [55]. However, in different tumors we can find opposite findings. Namely, Li et al. found that RIP5 expression was low in patients with lung adenocarcinoma and was associated with better prognosis [31]. Although both carcinomas were adenocarcinoma (kidney vs. lung), these difference in overall survival might be explained by a different biological function of RIPK5 that is largely unknown. The core kinase domains of RIP5 have multiple functional connections and distinct clinical properties in cancer, as supported by the data presented in the study by Li et al. [31].

Similarly, as involvement of RIP5 kinases in either pro-tumor or anti-tumor effect is largely unknown, VGLL4 has been identified as a tumor suppressor gene [56]. Therefore, due to its role as a tumor suppressor, VGLL4 has the potential to serve as a targeted therapy for various types of cancer. Namely, so far, VGLL4 has been investigated as a therapeutical target in triple-negative breast cancer, lung cancer, colorectal cancer, and gastric cancer [41,46,57,58]. Therefore, VGLL4 expression in mRCC also might represent an attractive therapeutic target.

The expression level of VGLL4 is lower in many types of cancer compared to normal tissues, and lower expression of VGLL4 usually indicates poor survival in many cancers [57]. VGLL4 is downregulated in ccRCC [56]. VGLL4 is also associated with metastasis and recurrence in ccRCC [59]. In our study, we found that in the renal cortex and medulla of normal control kidneys, VGLL4 is expressed in tubules with strong focal expression in epithelial cells. There was about one-quarter of VGLL4-positive cells in the control kidneys, which was statistically different only in comparison to grade 2. Additionally, the highest expression of VGLL4 was observed in grade 2, with more than two-thirds of positive cells in comparison to grade 3 and 4. VGLL4 displayed low expression in grade 4 mRCC, with few positive cells. In grade 3, there was about one-third of VGLL4-positive cells, with moderate expression and focal staining in the epithelial cells. Further, grade 3 displayed statistically higher expression in comparison to grade 4. The trend of linear decrease in the expression of VGLL4 was observed with the progression of tumor grade. Our results at the VGLL4 protein level are in accordance with the results on the transcriptomic level. Another two papers that investigated VGLL4 in ccRCC were performed on transcriptomic data and mRNA data in available open access databases [56,59]. Their findings also demonstrated worse overall survival in patients with low VGLL4 expression. This is in accordance with our findings.

The strength of our study is that this is the first study that evaluated RIP5 and VGLL4 protein expression by immunohistochemical staining in mRCC in different grades. Additionally, our results suggest that high mRNA expression of RIP5 and VGLL4 are important markers of better survival rates in mRCC patients. The limitation of our study is the small sample size, the retrospective design, and lack of in vitro effects of sunitinib treatment on RCC cell lines.

## 5. Conclusions

In conclusion, in this study, for the first time we presented the expression profiles of RIP5 and VGLL4 expression in mRCC that displayed involvement in different overall survival time and progression-free survival in mRCC. In this regard, our findings may contribute to efforts undertaken in identification of new treatment targets. However, the mechanistic role of RIP5 and VGLL4 in mRCC is an area that requires further study to fully elucidate its function in this specific context. Namely, further research into the pathogenesis, diagnosis, and management of mRCC is needed to continue improving treatment options for patients with this aggressive disease.

## Figures and Tables

**Table 1:** Primers used in RT-qPCR.

Transcript	Forward Primer	Reverse Primer	T_m_ F/R	T_A_	CG% F/R
RPL13a	CCTGGAGGAGAAGAGGAAAGAGA	TTGAGGACCTCTGTGTATTTGTCAA	63.1/60.5	60.5	52.17/40.00
RIP5	TTGCATACTGATCCTCGG	TGGCACTAGTTCATACT	59.3/55.8	55.8	50.0/38.89

**Table 2:** Demographic and clinical characteristics of patients.

	All Patients *n* = 34 n (%) or Median (Interquartile Range, IQR)
Gender Male	25 (74)
Age, years	61 (55–67)
Grade	
II	13 (38)
III	15 (44)
IV	6 (18)
pathological T	
T1	7 (21)
T2	3 (9)
T3	20 (59)
T4	4 (12)
pathological N	
N0	9 (26)
N1	3 (9)
Nx	22 (65)
pathological M	
M0	1 (3)
M1	0 (0)
Mx	33 (97)
TNM stage	
1	7 (21)
2	3 (9)
3	18 (53)
4	6 (18)
Presentation of metastatic disease—first year	
˂1 year	28 (82)
˃1 year	6 (18)
Metastasis site	
Lungs	26 (76)
Lymph nodes	7 (21)
Bones	9 (26)
Liver	4 (12)
Kidney	1 (3)
Brain	0 (0)
Other	5 (15)
Number of metastatic sites	
1	26 (76)
2	7 (21)
3	9 (26)
4	4 (12)
Overall death	
Yes	28 (82)
No	6 (18)
Distant metastasis-free survival	2.0 (0.3–7.0)
First-line progression-free survival	10.0 (1.3–20.1)
First-line overall survival	16.0 (1.3–24.0)

**Table 3:** Intensity and extension of staining for RIP5 and VGLL4 in different grades of mRCC (G2–G4) and control kidney (CTRL).

		RIP5	VGLL4
CTRL	epithelial cells	+++/>50%	+++/10–50%
	Stroma	−/<10%	−/<10%
G2	carcinoma cells	−/<10%	+/>50%
	Stroma	−/<10%	−/<10%
G3	carcinoma cells	+++/>50%	++/10–50%
	Stroma	−/<10%	+/<10%
G4	carcinoma cells	+++/>50%	−/<10%
	Stroma	−/<10%	−/<10%

− no expression; + mild expression; ++ moderate expression; +++ strong expression; no staining (<10% of epithelial/neoplastic cells); focal staining (10–50% of epithelial/neoplastic cells); diffuse staining (>50% of epithelial/neoplastic cells).

**Table 4:** RIP5 fold gene expression and survival in mRCC.

	RIP5 Fold Gene Expression ≤ 1 Mean Survival in Months (95% CI *) *n* = 19	RIP5 Fold Gene Expression > 1 Mean Survival in Months (95% CI *) *n* = 19	Log-Rank (Mantel–Cox) Test *p*-Value	Breslow (Generalized Wilcoxon) Test *p*-Value	Tarone–Ware Test *p*-Value
Overall survival	11.2 (4.0–18.4)	35.9 (17.7–54.1)	0.012	0.014	0.011
Progression-free survival	11.1 (3.6–18.7)	34.6 (15.0–54.1)	0.028	0.029	0.028
Distant metastasis free survival	9.6 (0.0–21.4)	18.7 (4.7–32.7)	0.246	0.166	0.172

* CI: confidence interval.

**Table 5:** Final Mantel–Cox proportional hazard regression model.

Covariate	Estimate	exp(Estimate) Hazard Ratio	Standard Error (Estimate)	95% Confidence Interval	*p*-Value
RIP5 gene expression group: more than one-fold compared to less than one-fold	−0.8591	0.4235	0.4012	0.193–0.930	0.0322

## Data Availability

Data will be available upon request.

## References

[1] Testa U., Pelosi E., Castelli G. (2020). Genetic Alterations in Renal Cancers: Identification of the Mechanisms Underlying Cancer Initiation and Progression and of Therapeutic Targets. Medicines.

[2] Padala S.A., Kallam A. (2020). Cancer, Clear Cell Renal Carcinoma. StatPearls.

[3] Sung H., Ferlay J., Siegel R.L., Laversanne M., Soerjomataram I., Jemal A., Bray F. (2021). Global Cancer Statistics 2020: GLOBOCAN Estimates of Incidence and Mortality Worldwide for 36 Cancers in 185 Countries. CA A Cancer J. Clin..

[4] Latif F., Duh F.M., Gnarra J., Tory K., Kuzmin I., Yao M., Stackhouse T., Modi W., Geil L., Schmidt L. (1993). von Hippel-Lindau syndrome: Cloning and identification of the plasma membrane Ca(++)-transporting ATPase isoform 2 gene that resides in the von Hippel-Lindau gene region. Cancer Res..

[5] Yao X., Tan J., Lim K.J., Koh J., Ooi W.F., Li Z., Huang D., Xing M., Chan Y.S., Qu J.Z. (2017). VHL Deficiency Drives Enhancer Activation of Oncogenes in Clear Cell Renal Cell Carcinoma. Cancer Discov..

[6] Zhang J., Wu T., Simon J., Takada M., Saito R., Fan C., Liu X.D., Jonasch E., Xie L., Chen X. (2018). VHL substrate transcription factor ZHX2 as an oncogenic driver in clear cell renal cell carcinoma. Science.

[7] Krishna C., DiNatale R.G., Kuo F., Srivastava R.M., Vuong L., Chowell D., Gupta S., Vanderbilt C., Purohit T.A., Liu M. (2021). Single-cell sequencing links multiregional immune landscapes and tissue-resident T cells in ccRCC to tumor topology and therapy efficacy. Cancer Cell.

[8] Du W., Zhang L., Brett-Morris A., Aguila B., Kerner J., Hoppel C.L., Puchowicz M., Serra D., Herrero L., Rini B.I. (2017). HIF drives lipid deposition and cancer in ccRCC via repression of fatty acid metabolism. Nat. Commun..

[9] Liu N., Gan W., Qu F., Wang Z., Zhuang W., Agizamhan S., Xu L., Yin J., Guo H., Li D. (2018). Does the Fuhrman or World Health Organization/International Society of Urological Pathology Grading System Apply to the Xp11.2 Translocation Renal Cell Carcinoma? A 10-Year Single-Center Study. Am. J. Pathol..

[10] Bai D., Feng H., Yang J., Yin A., Qian A., Sugiyama H. (2021). Landscape of immune cell infiltration in clear cell renal cell carcinoma to aid immunotherapy. Cancer Sci..

[11] Young J.R., Margolis D., Sauk S., Pantuck A.J., Sayre J., Raman S.S. (2013). Clear cell renal cell carcinoma: Discrimination from other renal cell carcinoma subtypes and oncocytoma at multiphasic multidetector CT. Radiology.

[12] Angulo J.C., Manini C., Lopez J.I., Pueyo A., Colas B., Ropero S. (2021). The Role of Epigenetics in the Progression of Clear Cell Renal Cell Carcinoma and the Basis for Future Epigenetic Treatments. Cancers.

[13] Hsieh J.J., Purdue M.P., Signoretti S., Swanton C., Albiges L., Schmidinger M., Heng D.Y., Larkin J., Ficarra V. (2017). Renal cell carcinoma. Nat. Rev. Dis. Primers.

[14] Nerich V., Hugues M., Paillard M.J., Borowski L., Nai T., Stein U., Nguyen Tan Hon T., Montcuquet P., Maurina T., Mouillet G. (2014). Clinical impact of targeted therapies in patients with metastatic clear-cell renal cell carcinoma. OncoTargets Ther..

[15] Rini B.I. (2009). Metastatic renal cell carcinoma: Many treatment options, one patient. J. Clin. Oncol. Off. J. Am. Soc. Clin. Oncol..

[16] Sorbellini M., Kattan M.W., Snyder M.E., Reuter V., Motzer R., Goetzl M., McKiernan J., Russo P. (2005). A postoperative prognostic nomogram predicting recurrence for patients with conventional clear cell renal cell carcinoma. J. Urol..

[17] Makhov P., Joshi S., Ghatalia P., Kutikov A., Uzzo R.G., Kolenko V.M. (2018). Resistance to Systemic Therapies in Clear Cell Renal Cell Carcinoma: Mechanisms and Management Strategies. Mol. Cancer Ther..

[18] Kotecha R.R., Motzer R.J., Voss M.H. (2019). Towards individualized therapy for metastatic renal cell carcinoma. Nat. Rev. Clin. Oncol..

[19] Ross K., Jones R.J. (2017). Immune checkpoint inhibitors in renal cell carcinoma. Clin. Sci..

[20] Shukla S., Robey R.W., Bates S.E., Ambudkar S.V. (2009). Sunitinib (Sutent, SU11248), a small-molecule receptor tyrosine kinase inhibitor, blocks function of the ATP-binding cassette (ABC) transporters P-glycoprotein (ABCB1) and ABCG2. Drug Metab. Dispos. Biol. Fate Chem..

[21] Motzer R.J., Hutson T.E., Tomczak P., Michaelson M.D., Bukowski R.M., Rixe O., Oudard S., Negrier S., Szczylik C., Kim S.T. (2007). Sunitinib versus interferon alfa in metastatic renal-cell carcinoma. N. Engl. J. Med..

[22] Molina A.M., Lin X., Korytowsky B., Matczak E., Lechuga M.J., Wiltshire R., Motzer R.J. (2014). Sunitinib objective response in metastatic renal cell carcinoma: Analysis of 1059 patients treated on clinical trials. Eur. J. Cancer.

[23] Crusz S.M., Tang Y.Z., Sarker S.J., Prevoo W., Kiyani I., Beltran L., Peters J., Sahdev A., Bex A., Powles T. (2016). Heterogeneous response and progression patterns reveal phenotypic heterogeneity of tyrosine kinase inhibitor response in metastatic renal cell carcinoma. BMC Med..

[24] Laccetti A.L., Garmezy B., Xiao L., Economides M., Venkatesan A., Gao J., Jonasch E., Corn P., Zurita-Saavedra A., Brown L.C. (2021). Combination antiangiogenic tyrosine kinase inhibition and anti-PD1 immunotherapy in metastatic renal cell carcinoma: A retrospective analysis of safety, tolerance, and clinical outcomes. Cancer Med..

[25] Zhang D., Lin J., Han J. (2010). Receptor-interacting protein (RIP) kinase family. Cell. Mol. Immunol..

[26] Kelam N., Racetin A., Polovic M., Benzon B., Ogorevc M., Vukojevic K., Glavina Durdov M., Dunatov Huljev A., Kuzmic Prusac I., Caric D. (2022). Aberrations in FGFR1, FGFR2, and RIP5 Expression in Human Congenital Anomalies of the Kidney and Urinary Tract (CAKUT). Int. J. Mol. Sci..

[27] Sanna-Cherchi S., Sampogna R.V., Papeta N., Burgess K.E., Nees S.N., Perry B.J., Choi M., Bodria M., Liu Y., Weng P.L. (2013). Mutations in DSTYK and dominant urinary tract malformations. N. Engl. J. Med..

[28] Racetin A., Raguz F., Durdov M.G., Kunac N., Saraga M., Sanna-Cherchi S., Soljic V., Martinovic V., Petricevic J., Kostic S. (2019). Immunohistochemical expression pattern of RIP5, FGFR1, FGFR2 and HIP2 in the normal human kidney development. Acta Histochem..

[29] Becic T., Kero D., Vukojevic K., Mardesic S., Saraga-Babic M. (2018). Growth factors FGF8 and FGF2 and their receptor FGFR1, transcriptional factors Msx-1 and MSX-2, and apoptotic factors p19 and RIP5 participate in the early human limb development. Acta Histochem..

[30] Bates C.M. (2011). Role of fibroblast growth factor receptor signaling in kidney development. Pediatr. Nephrol..

[31] Li G., Xu Z., Peng J., Yan Y., Liu Y., Zhang X., Qiu Y., Fu C. (2022). The RIPK family: Expression profile and prognostic value in lung adenocarcinoma. Aging.

[32] Zhang L., Guo W., Yu J., Li C., Li M., Chai D., Wang W., Deng W. (2021). Receptor-interacting protein in malignant digestive neoplasms. J. Cancer.

[33] Yin L., Duan J.J., Bian X.W., Yu S.C. (2020). Triple-negative breast cancer molecular subtyping and treatment progress. Breast Cancer Res..

[34] Nieto M.A., Huang R.Y., Jackson R.A., Thiery J.P. (2016). Emt: 2016. Cell.

[35] Chaffer C.L., San Juan B.P., Lim E., Weinberg R.A. (2016). EMT, cell plasticity and metastasis. Cancer Metastasis Rev..

[36] Lambert A.W., Pattabiraman D.R., Weinberg R.A. (2017). Emerging Biological Principles of Metastasis. Cell.

[37] Ermine K., Yu J., Zhang L. (2022). Role of Receptor Interacting Protein (RIP) kinases in cancer. Genes Dis..

[38] Vaudin P., Delanoue R., Davidson I., Silber J., Zider A. (1999). TONDU (TDU), a novel human protein related to the product of vestigial (vg) gene of Drosophila melanogaster interacts with vertebrate TEF factors and substitutes for Vg function in wing formation. Development.

[39] Maeda T., Chapman D.L., Stewart A.F. (2002). Mammalian vestigial-like 2, a cofactor of TEF-1 and MEF2 transcription factors that promotes skeletal muscle differentiation. J. Biol. Chem..

[40] Helias-Rodzewicz Z., Perot G., Chibon F., Ferreira C., Lagarde P., Terrier P., Coindre J.M., Aurias A. (2010). YAP1 and VGLL3, encoding two cofactors of TEAD transcription factors, are amplified and overexpressed in a subset of soft tissue sarcomas. Genes Chromosomes Cancer.

[41] Zhang W., Gao Y., Li P., Shi Z., Guo T., Li F., Han X., Feng Y., Zheng C., Wang Z. (2014). VGLL4 functions as a new tumor suppressor in lung cancer by negatively regulating the YAP-TEAD transcriptional complex. Cell Res..

[42] Zhang Y., Shen H., Withers H.G., Yang N., Denson K.E., Mussell A.L., Truskinovsky A., Fan Q., Gelman I.H., Frangou C. (2017). VGLL4 Selectively Represses YAP-Dependent Gene Induction and Tumorigenic Phenotypes in Breast Cancer. Sci. Rep..

[43] Jiao S., Wang H., Shi Z., Dong A., Zhang W., Song X., He F., Wang Y., Zhang Z., Wang W. (2014). A peptide mimicking VGLL4 function acts as a YAP antagonist therapy against gastric cancer. Cancer Cell.

[44] Li H., Wang Z., Zhang W., Qian K., Liao G., Xu W., Zhang S. (2015). VGLL4 inhibits EMT in part through suppressing Wnt/beta-catenin signaling pathway in gastric cancer. Med. Oncol..

[45] Li N., Yu N., Wang J., Xi H., Lu W., Xu H., Deng M., Zheng G., Liu H. (2015). miR-222/VGLL4/YAP-TEAD1 regulatory loop promotes proliferation and invasion of gastric cancer cells. Am. J. Cancer Res..

[46] Jiao S., Li C., Hao Q., Miao H., Zhang L., Li L., Zhou Z. (2017). VGLL4 targets a TCF4-TEAD4 complex to coregulate Wnt and Hippo signalling in colorectal cancer. Nat. Commun..

[47] Shivakumar M., Lee Y., Bang L., Garg T., Sohn K.A., Kim D. (2017). Identification of epigenetic interactions between miRNA and DNA methylation associated with gene expression as potential prognostic markers in bladder cancer. BMC Med. Genom..

[48] Liu X., Kong C., Zhang Z. (2018). miR-130b promotes bladder cancer cell proliferation, migration and invasion by targeting VGLL4. Oncol. Rep..

[49] Mann K.M., Ward J.M., Yew C.C., Kovochich A., Dawson D.W., Black M.A., Brett B.T., Sheetz T.E., Dupuy A.J., Chang D.K. (2012). Sleeping Beauty mutagenesis reveals cooperating mutations and pathways in pancreatic adenocarcinoma. Proc. Natl. Acad. Sci. USA.

[50] Jiang W., Yao F., He J., Lv B., Fang W., Zhu W., He G., Chen J. (2015). Downregulation of VGLL4 in the progression of esophageal squamous cell carcinoma. Tumour Biol..

[51] Veljacic Viskovic D., Lozic M., Vukoja M., Soljic V., Vukojevic K., Glavina Durdov M., Filipovic N., Lozic B. (2023). Spatio-Temporal Expression Pattern of CAKUT Candidate Genes DLG1 and KIF12 during Human Kidney Development. Biomolecules.

[52] Cohen H.T., McGovern F.J. (2005). Renal-cell carcinoma. N. Engl. J. Med..

[53] Davis M.I., Hunt J.P., Herrgard S., Ciceri P., Wodicka L.M., Pallares G., Hocker M., Treiber D.K., Zarrinkar P.P. (2011). Comprehensive analysis of kinase inhibitor selectivity. Nat. Biotechnol..

[54] Wodicka L.M., Ciceri P., Davis M.I., Hunt J.P., Floyd M., Salerno S., Hua X.H., Ford J.M., Armstrong R.C., Zarrinkar P.P. (2010). Activation state-dependent binding of small molecule kinase inhibitors: Structural insights from biochemistry. Chem. Biol..

[55] Zhong C., Chen M., Chen Y., Yao F., Fang W. (2021). Loss of DSTYK activates Wnt/beta-catenin signaling and glycolysis in lung adenocarcinoma. Cell Death Dis..

[56] Clark D.J., Dhanasekaran S.M., Petralia F., Pan J., Song X., Hu Y., da Veiga Leprevost F., Reva B., Lih T.M., Chang H.Y. (2019). Integrated Proteogenomic Characterization of Clear Cell Renal Cell Carcinoma. Cell.

[57] Deng X., Fang L. (2018). VGLL4 is a transcriptional cofactor acting as a novel tumor suppressor via interacting with TEADs. Am. J. Cancer Res..

[58] Song H., Luo Q., Deng X., Ji C., Li D., Munankarmy A., Jian W., Zhao J., Fang L. (2019). VGLL4 interacts with STAT3 to function as a tumor suppressor in triple-negative breast cancer. Exp. Mol. Med..

[59] Jiang A., Song J., Fang X., Fang Y., Wang Z., Liu B., Wu Z., Qu L., Luo P., Wang L. (2022). A novel thinking: DDR axis refines the classification of ccRCC with distinctive prognosis, multi omics landscape and management strategy. Front. Public Health.

